# Experimental and mouse-specific computational models of the *Fbln4*^SMKO^ mouse to identify potential biomarkers for ascending thoracic aortic aneurysm

**DOI:** 10.1007/s13239-021-00600-4

**Published:** 2022-01-22

**Authors:** Marisa S. Bazzi, Ramin Balouchzadeh, Shawn N. Pavey, James D. Quirk, Hiromi Yanagisawa, Vijay Vedula, Jessica E. Wagenseil, Victor H. Barocas

**Affiliations:** 1 Department of Chemical Engineering and Materials Science, University of Minnesota, Minneapolis, MN, 55455; 2 Department of Mechanical Engineering & Materials Science, Washington University, St. Louis, MO 63110; 3 Mallinckrodt Institute of Radiology, Washington University School of Medicine, St. Louis, MO 63110; 4 Life Science Center for Survival Dynamics, Tsukuba Advanced Research Alliance, University of Tsukuba, Japan; 5 Department of Mechanical Engineering, Columbia University, New York, NY 10027; 6 Department of Biomedical Engineering, University of Minnesota, Minneapolis, MN, 55455

## Abstract

The prediction of risk to the patient in ascending thoracic aortic aneurysm (ATAA) is a significant challenge and the subject of much active research. In the present work, a combination of mouse model experiments and computer simulations was used to explore potential biomarkers that correlate with mouse lifespan, used as a surrogate for risk of a catastrophic event. Image-based, mouse-specific fluid-structure-interaction models were developed for *Fbln4*^*SMKO*^ mice (n = 10) at ages two and six months. The results of the simulations were used to quantify potential biofluidic biomarkers, complementing the geometrical biomarkers obtained directly from the images. Comparing the different geometrical and biofluidic biomarkers to the mouse lifespan, it was found that mean oscillatory shear index (OSI_mean_) and minimum time-averaged wall shear stress (TAWSS_min_) at six months showed the largest correlation with lifespan (r^2^ = 0.70, 0.56), with both correlations being positive (i.e., mice with high OSI_mean_ and high TAWSS_min_ tended to live longer). When change between two and six months was considered, the change in TAWSS_min_ showed a much stronger correlation than OSI_mean_ (r^2^ = 0.75 vs. 0.24), and the correlation was negative (i.e., mice with increasing TAWSS_min_ over this period tended to live less long). The results highlight potential biomarkers of ATAA outcomes that can be obtained through noninvasive imaging and computational simulations.

## 1. Background:

Ascending thoracic aortic aneurysms (ATAAs) are bulging enlargements of the aorta at the ascending segment near the brachiocephalic trunk. They affect approximately 15,000 people in the US per year and, in critical cases, they will grow in size and eventually rupture. When an ATAA ruptures, it can be fatal in up to 80% of the cases [1]. Surgical intervention to replace the diseased region with a synthetic graft is the most common clinical treatment, but the surgical intervention itself presents risks, having a significant mortality rate (3-5%) [2]. The criteria to determine whether to perform surgery are defined primarily in terms of the maximum diameter and growth rate. About 60% of the patients, however, present complications before the intervention criteria are reached [3], indicating a need to improve the predictability efficiency for ATAA outcomes.

Numerous biomarkers [4]–[8] have been explored as a potential basis for predicting ATAA outcome. The biomarkers fall into different broad categories: genetic, microstructural, geometrical, and biofluidic. The primary genetic biomarkers are mutations known to lead to the formation of ATAA, such as Marfan syndrome [9] and Ehlers-Danlos syndrome [10]. Microstructural biomarkers are associated with alteration in the extracellular matrix proteins that lead to global or localized weakness of the arterial wall and have been associated with the formation and growth of ATAAs [11]. Geometrical biomarkers are the most well-known and are easily obtained from noninvasive imaging. They characterize changes in the overall geometry of the aorta, such as diameter and tortuosity [12]. Finally, biofluidic biomarkers are alterations in luminal pressure, wall shear stress, and intramural stress, that are associated with ATAA [13]. Biofluidic and geometrical biomarkers are particularly appealing because they are potentially noninvasive and patient-specific.

Although the ideal would be to use human subjects to identify the appropriate biomarkers and develop guidelines to interpret them, such experiments are impossible because of the ethical considerations regarding patient care and the long-life cycle of the human. Recent advances *in vivo* measurement of aortic wall mechanical properties [14] and availability of surgical waste tissue [15], [16] have provided some human data, but mouse models are more practical and offer the added benefit of tight control over genetic and environmental factors, hence they are an attractive tool to study ATAA [17].

The *Fbln4*^*SMKO*^ mouse model [18] was used in the current study. *Fbln4*^*SMKO*^ mice are genetically modified to eliminate expression of the gene encoding fibulin-4 in smooth muscle cells. FBLN4 mutations are associated with autosomal recessive cutis laxa type 1B (OMIM 614437) [19], so the mice have a known genetic biomarker for a disease associated with ATAA. Fibulin-4 is an extracellular matrix protein associated with microfibrils surrounding elastic fibers, and its deletion in smooth muscle cells in mice results in disorganized and fragmented elastic fibers, providing a known structural biomarker of ATAA [18]. *Fbln4*^*SMKO*^ mice develop ATAA postnatally, demonstrate aortic tortuosity [18], have altered biomechanics [17], [20], [21] and have an abbreviated lifespan [17], [18], providing an appropriate model to investigate geometrical and biofluidic biomarkers associated with adverse disease outcomes. Longitudinal images of the entire *Fbln4*^*SMKO*^ mouse aorta were captured at two different time points throughout disease progression. Those images were used, along with in vitro mechanical data, to build a mouse-specific fluid-structure interaction (FSI) model and to obtain aortic tortuosity and diameter measurements. The FSI models, mechanical properties, and aortic shape measurements were used to identify geometrical and biofluidic biomarkers that correlated with the mouse lifespan.

## 2. Methods:

### 2.1 Study design

**Figure 1.**
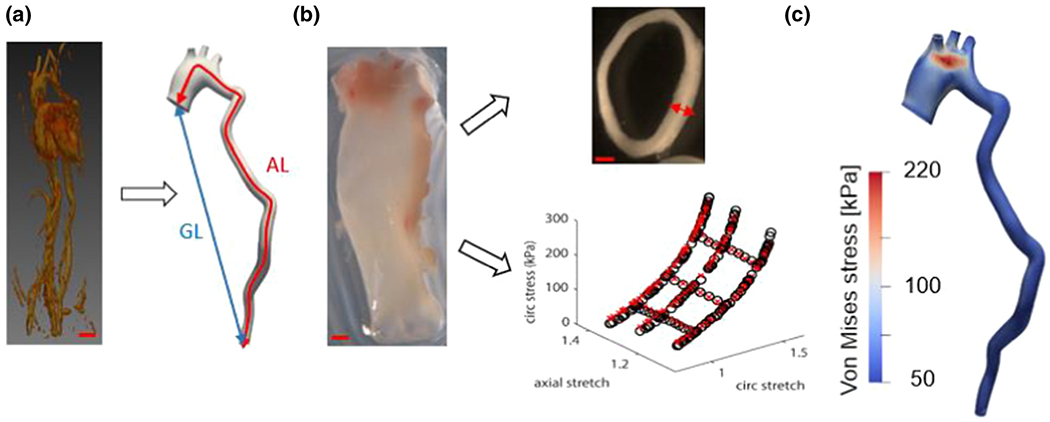
Summary of Models and Methods. *FBLN^SMKO^* mice [16] were used, which are known to exhibit elastic fiber fragmentation and ascending thoracic aortic aneurysm. **(a)** MRI scans were taken at ages two and six months (representative six-month scan of the heart and visible vasculature is shown), and the scans were used to generate geometric models of the aorta, from which the actual length (AL) and the geometric length (GL) were calculated. **(b)** After the animals died naturally or were euthanized, the aorta was isolated and used for mechanical testing, and rings were cut from the sample to allow measurement of wall thickness. **(c)** The geometric and mechanical data were combined to form the basis of age-specific, mouse-specific fluid-structure interaction models of aortic blood flow. Bars indicate 200 μm.

We used a combination of experimental and numerical techniques to capture changes in the geometry, mechanical properties, and fluid-solid dynamics during the development and formation of ATAA in mice. Figure 1 provides an overview of the study design. More details about each step are given in the following sections.

### 2.2 Genetically modified mice

Five male and five female mice lacking expression of fibulin-4 in smooth muscle cells (*Fbln4^SMKO^*) were used in this study [18]. Fibulin-4 is critical for elastic fiber assembly, and *Fbln4^SMKO^* mice have ATAAs with about 50% penetrance. Mice were monitored regularly; deceased mice were immediately collected, and the thoracic aorta was removed for mechanical testing. Any mice still alive at 25 months of age were euthanized by CO_2_ inhalation, and the thoracic aorta was removed for mechanical testing. All animal procedures were approved by the Institutional Animal Care and Use Committee at Washington University.

### 2.3 Magnetic resonance imaging (MRI)

Mice were imaged at two and six months of age. For the imaging procedure, mice were anesthetized with 1-2% isoflurane and imaged in the supine position, attached to ECG leads, and imaged on an Agilent DirectDrive 4.7T MRI (Agilent Technologies, Santa Clara, CA) with a 3.5 cm birdcage radiofrequency coil. Axial 2D gradient echo cine images at 21 cardiac phases were acquired using prospective cardiac and respiratory gating. The imaging parameters were as follows: field of view = 2.4 x 2.4 cm, imaging matrix 128 x 128, echo time 1.3 ms, the repetition time was determined by the cardiac rate, flip angle = 90 degrees, 70-80 slices at 0.5 mm thickness covering the aortic arch through the renal arteries (acquired sequentially in groups of 10 to account for variation in cardiac rate). The acquired images had a resolution of 187.5 x 187.5 x 500 μm^3^. The images corresponding to diastole were averaged and interpolated to 250 μm slice thickness for further analyses. Representative images of axial slices (Figure S1) and a coronal maximum intensity projection (Figure S2) are included in the supplementary data.

Geometrical parameters were determined from the MR images using MATLAB (Mathworks, Natick, MA). The aorta was segmented from the axial image stacks from the aortic root to the bifurcation. The outer surface of the aortic lumen was identified, and circles were fit perpendicular to the outer surface. The centroid and diameter (D) of each circle were identified, as well as the actual length (AL = length following the aortic contour of the fitted centroids) and geometric length (GL = straight-line length between centroids), as seen in Fig. 1a. The maximum diameter was calculated from the fitted circles and the aortic tortuosity index (ATI) was calculated as (AL/GL-1) x 100[22].

### 2.4 Mechanical testing

The ascending and descending aorta were removed for mechanical testing (Fig 1b), as previously [23], [24]; for the present analysis, only the ascending aorta data were used. Briefly, the aorta was mounted in a pressure myograph (DMT USA, 110P) in physiologic saline at 37°C and preconditioned for at least three cycles in each direction (circumferential and axial). The aorta then underwent six mechanical testing protocols: three cyclic inflation cycles at a constant axial stretch (0 – 175 mmHg pressure at approximately 1.3, 1.4, and 1.5 axial stretches) and three cyclic axial stretch cycles (from approximately 1.3 – 1.5 axial stretches at 50, 100, and 150 mmHg pressures). Details on the methods are presented elsewhere [23], [24]. Thin rings were cut from the aorta after testing, and images were taken to measure the unloaded diameter and thickness (Fig 1b).

### 2.5 Aortic wall constitutive equation

To account for the anisotropic and nonlinear mechanical behavior of the arterial wall, we described the tissue using the Holzapfel, Gasser, and Ogden (HGO) model [25] for the strain energy density function.

(1) $$\text{Ψ}=\frac{c}{2}\left(I_{1}-3\right)+\overset{2}{\underset{i=1}{\sum }}\frac{k_{1}}{k_{2}}\left[\exp\left\{k_{2}{\left[\kappa I_{1}+(1-3\kappa )I_{4i}-1\right]}^{2}\right\}-1\right]$$

The first term of Eq. 1 represents an isotropic neo-Hookean matrix, the quantity *I*_1_ = tr(***F***^*T*^ ***F***) is the first invariant of ***F***^*T*^ ***F***, where ***F*** is the deformation gradient tensor, and *c* denotes the neo-Hookean parameter. The second term accounts for the anisotropic behavior of a fibrillar component comprising two fiber families. The invariant $I_{4i}=\lambda_{\theta }^{2}\;\cos^{2}\;\gamma_{i}+\lambda_{z}^{2}\;\sin^{2}\;\gamma_{i}$ describes the stretch along the fiber direction *γ_i_*, where *λ_θ_* is the tissue stretch ratio along the circumferential direction, and *λ_z_* is the stretch ratio along the axial direction. The parameter *κ* characterizes the fiber dispersion, *k*_1_ > 0 is a modulus-like parameter, and *k*_2_ > 0 is a dimensionless nonlinearity parameter.

It was assumed that *γ*_1_ = −*γ*_2_ = *γ*, and that *k*_1_, *k*_2_, and *κ* were the same for both fiber populations, leaving five parameters (c, *k*_1_, *k*_2_, *κ*, and *γ*) that were fit for each mouse using MATLAB. The experimental data for the ascending aorta region were used since most of the aneurysms were formed in that region. The fitting was obtained by minimizing the mean squared error between the theoretical and the experimental circumferential and axial stresses. Assuming incompressibility and negligible shear, the wall stresses were calculated as follows:

(2) $$\sigma_{z}=\lambda_{z}\frac{\partial \text{Ψ}}{\partial \lambda_{z}}$$

(3) $$\sigma_{\theta }=\lambda_{\theta }\frac{\partial \text{Ψ}}{\partial \lambda_{\theta }}$$

Two out of the ten *Fbln4^SMKO^* mice did not have the mechanical or unloaded geometry data available, so for those two mice, we estimate the parameters by using the average data for the other eight mice. An elastic modulus (E) was calculated from the isotropic contribution of HGO model, by using the relation E=c(1+ν), where ν is the Poisson’s ratio of the tissue. This modulus, although not used in the computations, provided a rough measure of the overall tissue stiffness.

### 2.6 Mouse-specific FSI simulations:

#### 2.6.1 Model construction and mesh generation

FSI models were constructed for each mouse using two- and six-month MRI scans. Two domains are required for the FSI simulations: the fluid domain, given by the vessel lumen, and the solid domain, representing the aortic wall. A 3D model of the fluid domain was generated from MRI scans. Scans were segmented using image segmentation and model generation in SimVascular[26], with supplemental editing in Meshmixer (Autodesk, Inc.). The 3D model included the brachiocephalic trunk, the left common carotid, and the left subclavian artery.

Because the aortic wall is too thin to measure directly from the MR images, the aortic wall was generated by extruding the lumen wall outward. The mouse-specific unloaded wall thickness was based on experimental measurement for the ascending aortic region shown in Table 1 and was treated as constant over the length of the aorta.

A tetrahedral mesh was created using the TetGen mesh generator that is embedded in SimVascular [27]. The mesh included both the solid and the fluid domains, with matching nodes at the interface between the domains to satisfy the kinematic and dynamic boundary conditions at the interfaces.

#### 2.6.2 Boundary conditions

For the solid domain, a fixed boundary at the inlet and outlets was assumed. For the outer boundary of the arterial wall, to account for the fact that the aorta is surrounded by various tissues and organs that restrict the aorta movement and dilation, a Robin-type boundary condition was imposed [28]:

(4) $$\sigma_{s}\cdot n=-k_{s}u-c_{s}\frac{\partial u}{\partial t}-p_{0}n$$

Where ***σ***_*s*_ · ***n*** is the traction arising when the Cauchy stress tensor ***σ***_*s*_ at the wall is projected along the normal direction ***n***. The variable ***u*** is boundary displacement, and $\frac{\partial u}{\partial t}$ is the local tissue velocity. Parameters *k_s_* and *c_s_* account for the viscoelastic response of the external tissue, and *p*_0_ is the external pressure of the abdominal and thoracic cavity. Parameters values were set as follow: *k_s_* = 10^7^ N.s/m^3^, *c_s_* = 10^4^ N.s^2^/m^3^, and *p*_0_ = 0 Pa. Those values are in the range of the values reported in the literature [28], [29].

**Figure 2:**
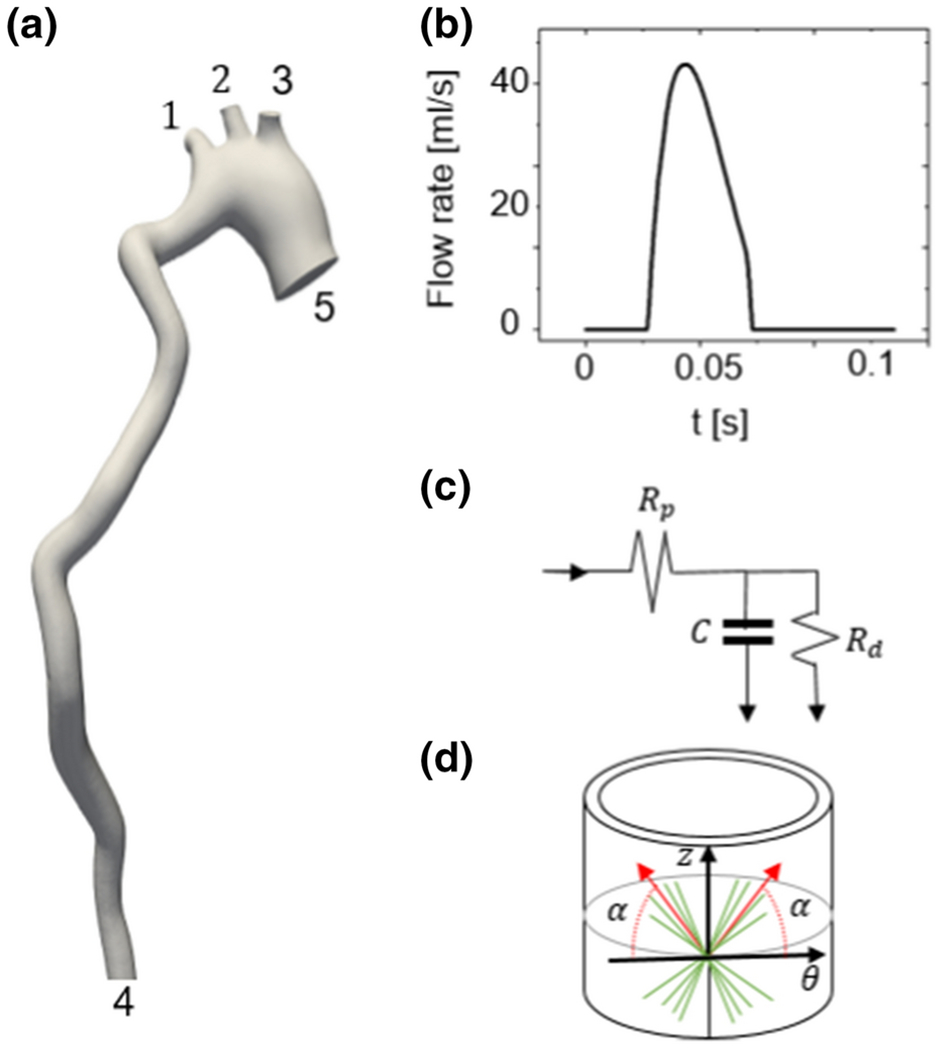
**(a)** Summary of the boundary conditions for FSI simulations including: **(b)** Inflow flow rate waveform for inlet at location 5. **(c)** Three-element windkessel model for outlets at locations 1-4, values are giving inn Table 2. **d)** Anisotropic hyperelastic material model for the wall described in Eq.1 that accounts for 2 fiber families oriented with an angle *α*

The fluid domain boundary conditions are shown in Fig 2. A waveform from the mouse study of Cuomo et al. [30] (Fig 2b) was used as the inlet boundary condition and was assumed to be the same for all mice. For the outlet arteries, a three-element circuit analog model (Fig. 2c) was prescribed as boundary conditions.

(5) $$\frac{\partial p}{\partial t}+\frac{p}{R_{d}C}=\frac{Q}{C}\left(1+\frac{R_{p}}{R_{d}}\right)+R_{p}\frac{\partial Q}{\partial t}$$

where *p* is the spatially averaged pressure at each outlet, *Q* is the inflow rate at each outlet, *R_d_* is the distal resistance, *R_p_* is the proximal resistance, and *C* is the compliance of the downstream vasculature. The values for *R_d_*, *R_p_*, and *C* were based on literature [30] and are given in Table 2.

#### 2.6.3 Blood rheology

Although non-Newtonian behavior is often neglected in a large artery, blood flow in the mouse aorta presents a Reynolds number of the order of 200 [31], which is much lower than the values observed in humans (~1000) [32]. Consequently, the shear stress may not be large enough for blood to be treated as a Newtonian fluid. To account for the non-Newtonian behavior of the mouse blood, we describe the blood viscosity using the shear-thinning Carreau-Yasuda model:

(6) $$\eta =\eta_{\infty }+\left(\eta_{0}-\eta_{\infty }\right){\left(1-{\left(\lambda \dot{\gamma }\right)}^{a}\right)}^{\frac{n-1}{a}}$$

where *η* is the apparent viscosity as a function of the shear rate $\tilde{\gamma }$. *η*_∞_ is the infinite-shear-rate viscosity, which describes the low-viscosity plateau region for high shear rate flow conditions. The zero-shear-rate viscosity *η*_0_ describes the high-viscosity plateau region for low shear rate flow conditions. The parameters *a*, *n*, and *λ* describe the power-law region between the two plateaus. Parameter values were based on our previous analysis of healthy human blood [33], which is similar to mouse blood rheology for the shear rate range of the interest [34] (Supplemental Table S1): *η*_∞_ = 2 *cP*, *η*_0_ = 11 *cP*, *λ* = 1.5, *a* = 0.2, *n* = 0.71.

#### 2.6.4 Prestress of the structural domain

*In vivo*, the aortic wall is subjected to mechanical load from the blood pressure, even during diastole. Accounting for this underlying mechanical loading state is crucial to get an accurate material response during FSI, especially when the model is constructed from *in vivo* image acquisition. In particular, Baumler *et al.* [35] showed that the prestress helps to reduce the numerical artifacts such as deviations in the aortic diameter and the drop in diastolic pressure.

We used the approach proposed by Hsu and Bazilevs [36] and embedded it in the FSI solver of SimVascular [37], [38]. A prestress tensor is determined via a three-step process: 1) An approximation for the diastolic load exerted on the aortic wall is derived based on the traction obtained from rigid-wall blood flow simulation for the fluid domain under diastolic inflow rate and using the same boundary conditions as in the FSI simulation, 2) the balance of momentum between the solid domain and the fluid traction is used to obtain the prestress tensor, and 3) the prestress tensor is used for the FSI simulations.

Once the prestress had been determined, dynamic simulations were performed for ten cardiac cycles using the svFSI solver from SimVascular [39] at the Minnesota Supercomputer Institute (MSI) using 120 CPUs for about sixty hours.

### 2.7 Data analysis and statistics:

#### 2.7.1 Tobit model

Four out of the ten mice were euthanized at age 25 months, censoring the data at a maximum of 25 months. To account for the censored data, we used the Tobit model [40] given by Eq. 7.

(7) $$Y_{t}=X_{t}\beta +u_{t}\;\;\;\;\text{if}\;\;X_{t}\beta +u_{t}<Y_{\max}\;Y_{t}=Y_{\max}\;\;\;\;\text{if}\;X_{t}\beta +u_{t}\geq Y_{\max}$$

In this model *Y_t_* is the dependent variable, in our case, the mouse lifespan; *X_t_* are the independent variables, being fluid dynamics and geometrical biomarkers; *β* is the fitting coefficient; and *Y_max_* is the censor cut off, 25 months in our case. The stochastic error, *u_t_* is assumed to have a normal distribution, mean at zero, and a constant variance *σ*^2^. Here *t* = 1,2, …, *N*, with N being the number of observations.

Since the Tobit model limits the dependent variable, the traditional r^2^ is not the best measurement to evaluate the goodness-of-fit. We used the modified McKelvey and Zavoina pseudo-r^2^ [41] since it has been shown to recover the standard r^2^ values for non-censored data.

(8) $$r^{2}=\frac{\sum_{i=1}^{N}{\left(Y_{t}-{\bar{Y}}_{t}\right)}^{2}}{\sum_{i=1}^{N}{\left(Y_{t}-{\bar{Y}}_{t}\right)}^{2}+N\sigma^{2}}$$

where ${\bar{Y}}_{t}$ is the mean of *Y_t_*.

#### 2.7.2 Correlation analysis

A correlation map was built using selected fluid-dynamical, geometrical, and mechanical biomarkers. The list of the biomarkers used in this study is given in Table 3. Here, Δ represents the absolute difference between the parameters’ values from two to six months. Maximum, minimum, and mean values are calculated throughout the tenth cardiac cycle and in the whole aorta. Parameters calculated at six months of age were considered possible biomarkers for lifespan, as was the change in the parameter values from two to six months of age for an individual mouse. $Re_{\max}=\frac{\rho V_{\max}D_{\max}}{\mu_{avg}}$ was calculated using maximum velocity *V_max_* at the peak of the 10^th^ cardiac cycle, and the maximum aortic diameter *D_max_*. The blood density was assumed to be *ρ* = 1027 kg/m^3^. The average viscosity *μ_avg_* = 3.35 *cP* was calculated for the physiological murine shear rate range. The time average wall shear stress (TAWSS) is the average of the wall shear stress (WSS) during a full cardiac cycle:

(9) $$\mathrm{TAWSS}=\frac{1}{T}\int_{0}^{T}|\tau |dt$$

where T is one cardiac cycle, and $\left|\vec{\tau }\right|$ is the magnitude of the WSS. The oscillatory shear index (OSI) accounts for the direction change in the WSS vector $\vec{\tau }$ during the cardiac cycle:

(10) $$OSI=\frac{1}{2}\left(1-\frac{\left|\int_{0}^{T}\;\tau dt\right|}{\int_{0}^{T}|\tau |dt}\right)$$

OSI ranges from 0 to 0.5, with an OSI of 0 meaning that ***τ*** does not change during the cardiac cycle and an OSI of 0.5 meaning that ***τ*** fluctuates in 180°. The pseudo–r^2^ given by Eq. 8 and based on the Tobit model fit Eq. 7 was calculated for each biomarker.

## 3. Results:

### 3.1 Experimental measurements and mechanical model

#### 3.1.1 Mechanical model fitting and analysis

**Figure 3:**
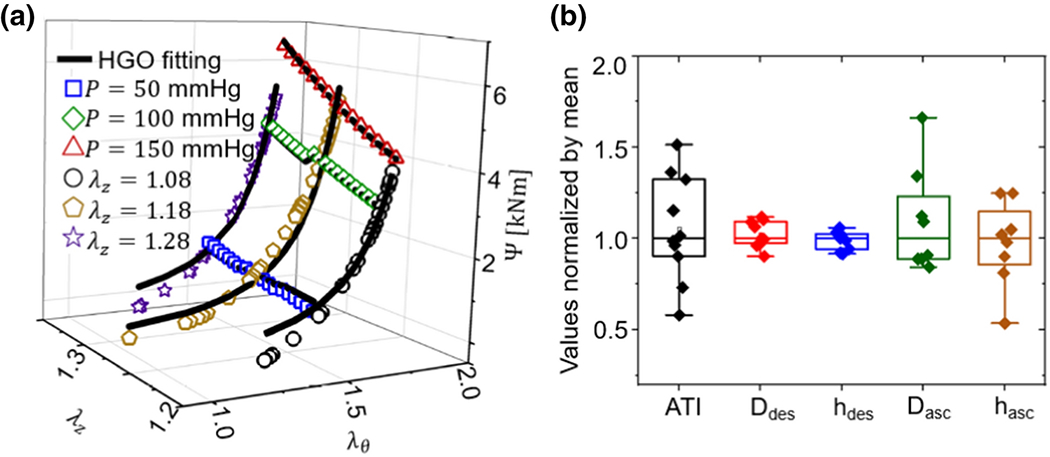
**(a)** Strain energy density for different experimental protocols (individual symbols) and the fitted HGO model (solid line) for a representative aorta. **(b)** Geometrical measurements normalized by mean, where means are: ATI = 39.2, D_desc_ = 0.87 mm , h_desc_ = 0.12 mm, D_asc_ = 1.53 mm, h_asc_ = 0.16 mm

We fitted the mechanical data for the ascending the aorta of each *Fbln4^SMKO^* mouse separately using the HGO model of Eq. 1. Figure 3a shows the experimental and fitted strain energy density function for a representative sample over the range of loading conditions. Results show a good agreement between experimental measurements and the material model.

Table 4 shows the summary data of fitting parameters for all eight mice. High standard deviations are observed for all the parameters, suggesting widely different mechanical behavior among the mice.

Geometrical biomarkers obtained experimentally, such as aortic tortuosity index (ATI) for 6-month *Fbln4^SMKO^* mice, and post-mortem diameter (D) and thickness (h) for the ascending aorta were also analyzed. Although the descending aorta data were not treated as potential biomarkers, they were obtained and are shown in Fig. 3b for comparison, along with the other geometric data, all normalized by the mean. Mean values and standards deviation are: *ATI* = 39.2 ± 11.4, D_desc_ = 0.87 ± 0.065 mm, h_desc_ = 0.12 ± 0.0062 mm, D_asc_ = 1.59 ± 0.38 mm, h_asc_ = 0.16 ± 0.043 mm. As can be seen in Fig. 3b, there was a considerable variation in ATI, and descending aortic geometry (D_des_, h_des_) was much more stable across mice than ascending aortic geometry (D_asc_, h_asc_). This difference was to be expected since the aneurysms were present in the ascending aortic region.

The fitted parameters for the tissue material properties were used as input for FSI simulations. The geometrical data is used directly as biomarkers and potential predictors for aneurysm outcomes.

### 3.2 Mouse-specific FSI simulations

**Figure 4:**
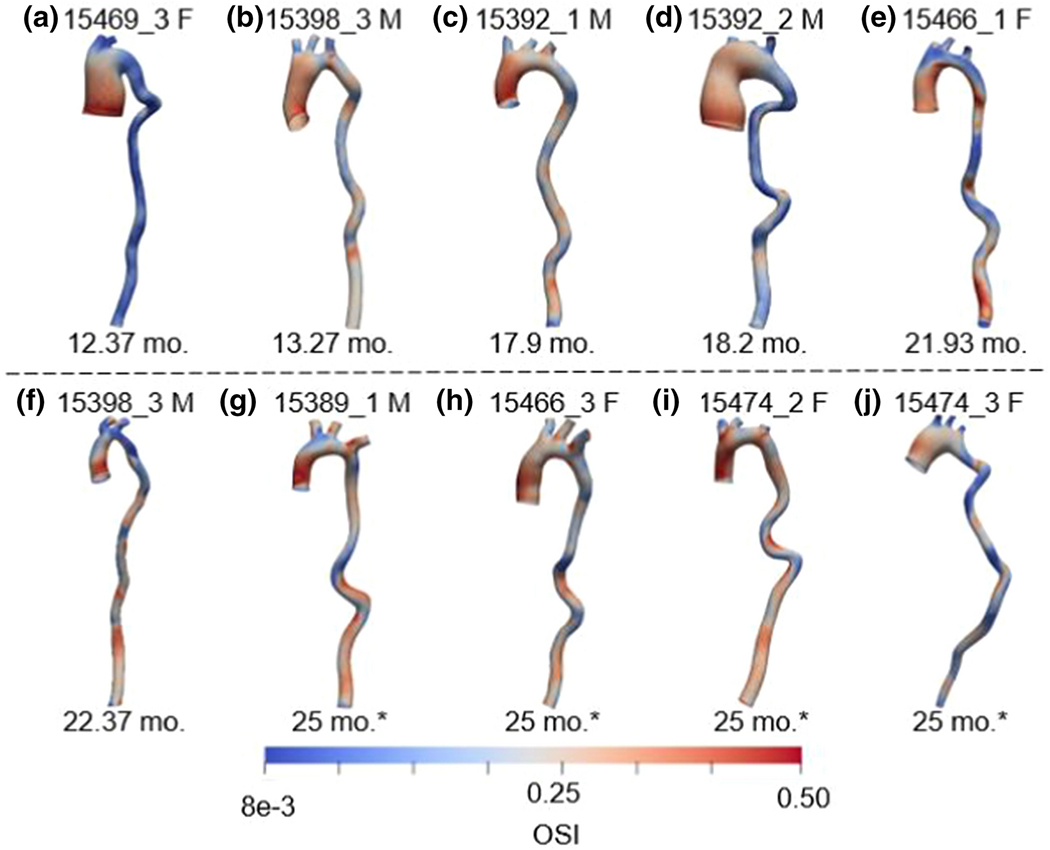
OSI distribution along the wall for all ten *Fbln4^SMKO^* mice. Models are organized by increasing lifespan. Mouse ID code and sex (M/F) is given at the top of each model, and lifespan is displayed on the bottom. Asterisks refer to mice that were euthanized at 25 month age.

Figure 4 shows the Oscillatory Shear Index (OSI) distribution along the wall for all ten six-month-old *Fbln4^SMKO^* mice at the 10^th^ simulated cardiac cycle. The geometrical differences among the mice are easily seen, with a wide range of aneurysm size and shapes at this age, as well as a large spectrum of tortuosity in the descending thoracic aorta. For the OSI, higher values were observed close to tortuous regions in the descending aorta, and in the ascending aorta and arch, especially for large aneurysms. *Fbln4^SMKO^* Mice exhibiting large aneurysms (e.g. Fig. 4a and Fig. 4d) presented a lower OSI along the descending aorta. Time average wall shear stress (TAWSS) for each individual aorta is shown in the supplemental information (Figure S3).

### 3.3 Sensitivity analysis on the material properties and blood rheology

**Figure 5:**
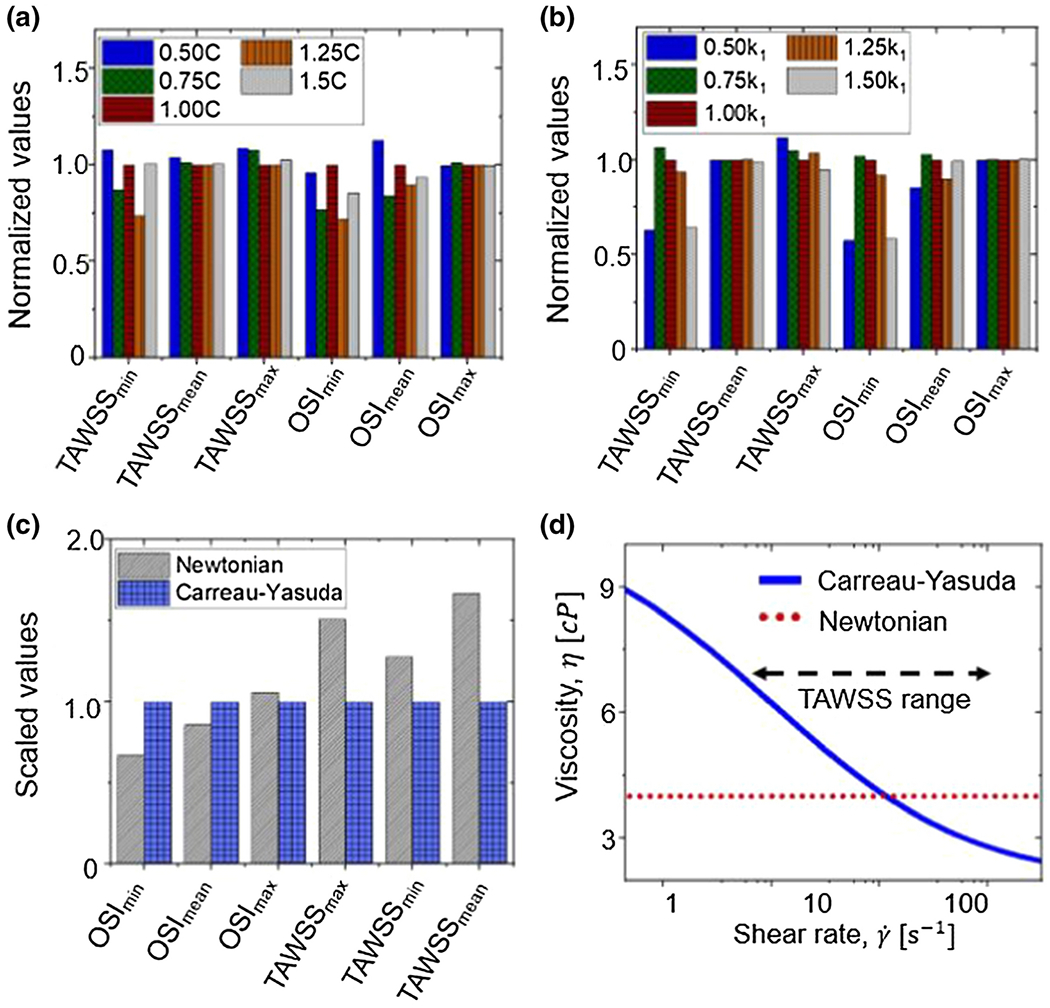
Sensitivity analysis for **(a)** Parameter *c* of the HGO model (Eq. 1) normalized by the fitted value 1.00*c* **(b)** parameter *k*_*1*_ of the HGO model (Eq. 1) normalized by the fitted value 1.00*k_1_* **(c)** blood rheology comparing Carreau-Yasuda and Newtonian approximation normalized by the Carreau-Yasuda results. **(d)** Viscosity versus shear rate for Carreau-Yasuda model (solid line) and Newtonian model (dashed line)

**Figure 6:**
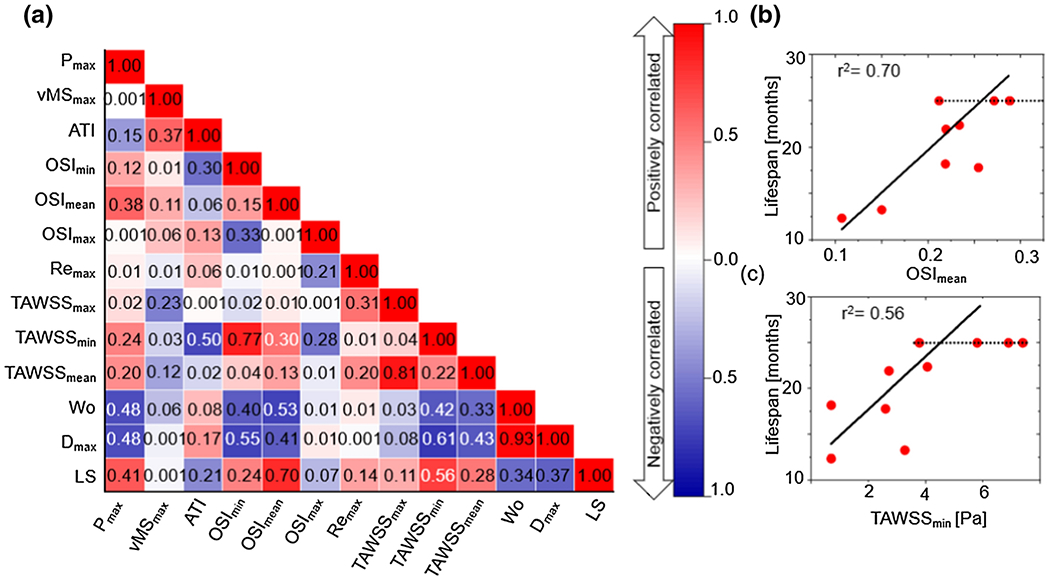
**(a)** Correlation map for biofluidic and geometrical biomarkers for six-month-old *Fbln4^SMKO^* mice. The numbers in the map are the pseudo-r2 values based on equation (8), and the colors indicate positive (red) vs. negative (blue) correlation, with darker colors signifying a stronger correlation. **(b)** Mean OSI and lifespan correlation plot. The solid line is the Tobit model fit, and the dashed line is the censoring cut-off. **(c)** Minimum TAWSS and lifespan correlation plot.

A sensitivity analysis was performed to evaluate the impact of the material properties and fluid rheology on the results. For the material property analyses, four different variations of the modulus-like parameters (*c*, *k_1_* from Eqn. 1) for one *Fbln4^SMKO^* mouse were explored: 50% and 25% smaller and 25% and 50% larger than the fitted values. Figure 5 shows the results of the analysis. For the parameter *c* (Fig 5a) and parameter *k_1_* (Fig 6b) of the HGO model presented in Eq. 1, the results suggest that moderate changes in the parameters (25% increase or decrease) have little or no impact on the results of the blood flow dynamics biomarkers.

For the fluid rheology analyses, a Carreau-Yasuda model was compared against the Newtonian approximation. Figure 5c shows a substantial change in all the parameters for the Carreau-Yasuda model in contrast to the Newtonian approximation. This effect could have occurred because the biomarkers used in the analysis are sensitive to changes in velocity. Close to the wall, the shear rate is moderately high (~350 1/s) leading to a drop in the viscosity as shown in Fig 5d.

### 3.4 Statistical analysis

Taking the results obtained from the FSI simulations, we explored the relevance of each biofluidic, geometrical, and mechanical biomarker as a predictive indicator for the outcome of ATAA. We used mouse lifespan as a surrogate indicator for ATAA outcomes, assuming that reduced lifespan was due to complications associated with ATAA.

Figure 6a shows a pseudo-r^2^ correlation matrix for the results from the FSI simulation and experimental measurements. The bottom row highlights the correlation or lack thereof between the main biomarkers and the mouse lifespan. Red indicates a positive correlation meaning that mice tend to live longer with an increase in the parameter. Conversely, blue indicates a negative correlation.

Among these, OSI_mean_ showed the highest positive correlation (r^2^=0.70) to the mouse lifespan, followed closely by TAWSS_min_ (r^2^ = 0.56), as shown in more detail in Figs 6b and c. Note that the minimum and mean OSI calculations are dominated by the contribution of the descending aorta (Figure 4), meaning that a low OSI_mean_, which corresponded to a shorter lifespan, indicated less oscillatory flow in the descending aorta, well downstream of the diseased segment and the presumed ultimate site of tissue failure. A low OSI downstream would be consistent with greater flow damping by the ascending aorta, which would result from a larger aneurysm with resulting greater damping capacity. In contrast TAWSS_min_ was generally observed in the ascending aorta (Supplemental Figure S3), suggesting that, unlike OSI_mean_, it is acting as a direct biomarker of biofluidic events in the aneurysmal region.

**Figure 7:**
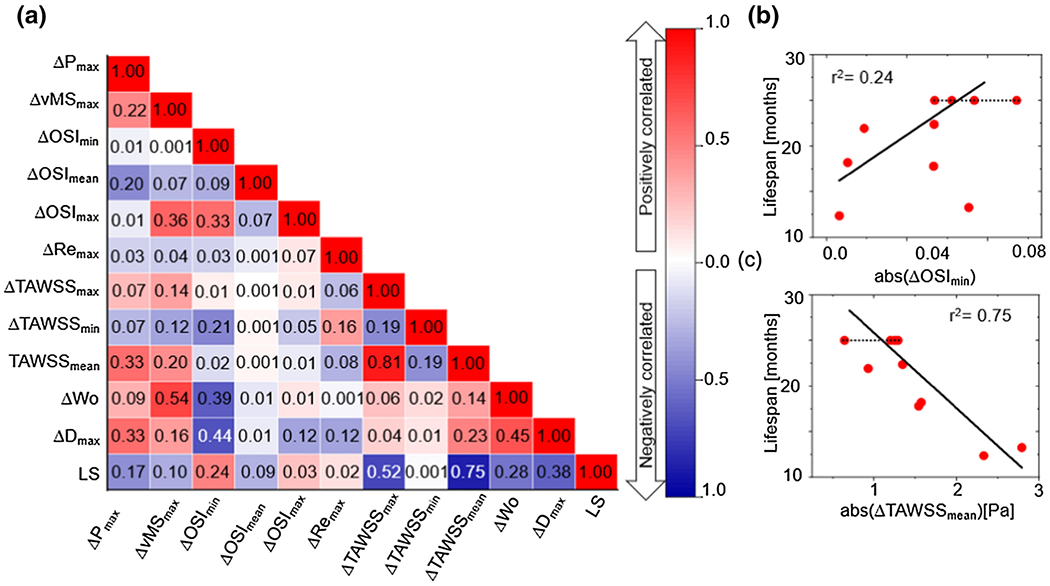
**(a)** Correlation map for temporal changes in biofluidic and geometrical biomarkers from 2 to 6 months of age in *Fbln4^SMKO^* mice. The numbers in the map are the pseudo-r2 values based on equation (8), and the colors indicate positive (red) vs. negative (blue) correlation, with darker colors signifying a stronger correlation, **(b)** Minimum OSI and lifespan correlation plot. **(c)** Mean TAWSS and lifespan correlation plot.

We also investigated the change from 2 to 6 months of age in the biofluidic, geometrical, and mechanical biomarkers and their correlation to the *Fbln4^SMKO^* mouse lifespan. Figure 7a shows the correlation matrix for the absolute change in each biomarker between two- and six-month-old mice. Similar to the single-time-point analysis, ΔOSI and ΔTAWSS have some of the highest correlations with mouse lifespan, however the value that correlates with lifespan in each case is different. For the change in values over time, ΔOSI_min_ has a moderate positive correlation with lifespan (r^2^=0.24, Fig. 7b), while ΔTAWSS_mean_ has a high negative correlation with lifespan (r^2^=0.75, Fig. 7c), followed closely by ΔTAWSS_max_ (r^2^=0.52). These results indicate that longitudinal monitoring of biofludic biomarkers, such as TAWSS, may provide additional predictive information about ATAA outcomes.

**Figure 8:**
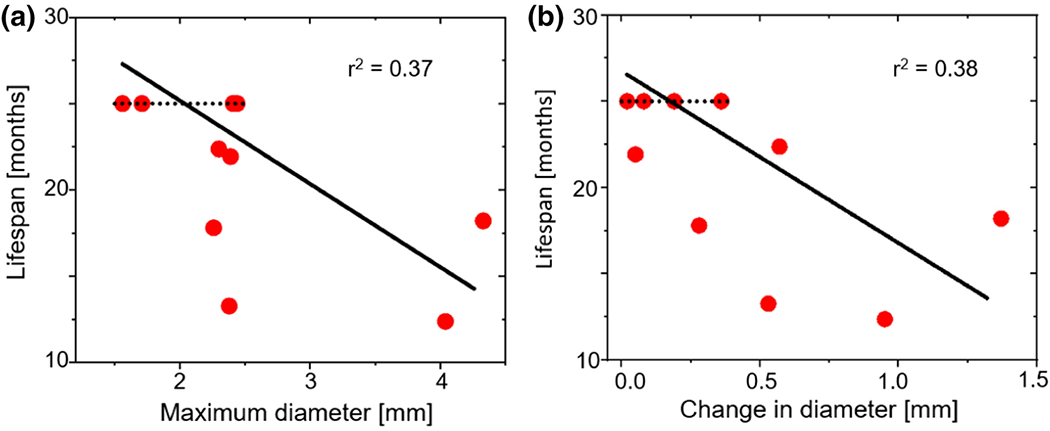
**(a)** Maximum aortic diameter and lifespan correlation plot. **(b)** Aortic diameter change from two to six month of age and lifespan correlation plot.

Figure 8 shows the correlation plot for correlation between the maximum diameter (D) and the lifespan (LS) and change in the diameter (ΔD) and the lifespan. Both show a negative correlation with lifespan, which is expected. There was a moderate correlation for both cases: r^2^=0.37 for diameter and lifespan, and r^2^=0.38 for change in diameter and lifespan. Although the maximum diameter and temporal change are clinical criteria for the surgical treatment of patients, our results support our argument for additional and more accurate biomarkers for predicting ATAA outcomes. In fact, previous work has shown that diameter and change in diameter criteria fail to predict an aneurysm rupture event, especially with patients who have presented prior dissection of the aorta [3].

## Discussion

The most important contribution of this work is its use of combined experimental and computational techniques to study the temporal evolution of ATAA in ten *Fbln4^SMKO^* mice. The combined approach allowed us to extract geometrical and mechanical data for each mouse to build mouse-specific FSI simulations, and use the results to study the predictive capacity of non-invasive biomarkers of aneurysm disease.

Mouse-specific blood flow models are not novel per se [42]–[45], and [42] in particular provides a thorough description of the advantages and challenges of applying computational fluid dynamics to small animals. The novelty of the current work is in (1) its specific application to ATAA, (2) its use of post-mortem biomechanical data to construct an FSI model with each mouse’s specific aortic wall thickness and mechanical properties, (3) its use of computational analyses based on two different imaging time points, and (4) its direct comparison of biomarkers to mouse lifespan without intervention, an obviously revelant metric of long term outcomes of ATAA that cannot ethically be used in humans. Although the sample size was small (N = 10), certain key results arose that could help guide future animal and human studies.

The strongest correlation of a single measurement with lifetime was for the calculated OSI_mean_ (r^2^ = 0.70, Fig. 6b), followed by the TAWSS_min_ (r^2^=0.56, Fig. 6c). These two quantities, which can be readily calculated from simulations based on human patient images [46], [47], showed considerably more correlation with lifetime than aneurysm diameter (r^2^ = 0.37, Fig. 8a), which is routinely used to access risk. TAWSS [48], [49] and OSI [50], [51] have both been associated with arterial remodeling, although the impact of these factors on ATAA has not been defined conclusively. Some studies [46], [52]–[54] suggest that low WSS values are related to wall weakening and dilation, whereas another study [55] found low WSS to be associated with decreased ATAA rupture risk. In the current study, we found that TAWSS_min_ had a positive correlation with lifespan, meaning that a lower value of TAWSS_min_ was associated with a shorter lifespan. The FSI simulations showed low TAWSS in the aneurysmal region (Supplemental Figure S3), consistent with TAWSS being an important potential biomarker.

We found that OSI_mean_ had a positive correlation with lifespan, indicating that a higher mean oscillatory flow correlated with longer life. Similar to WSS, the effect of OSI on aortic rupture is controversial and not yet fully understood. Some studies suggest that high OSI values are associated with a high risk of aortic rupture [56], [57], while others argue that OSI has no effect on rupture risk [58]–[60]. It may also be important to consider where OSI is being measured. In our simulations, OSI in the descending thoracic aorta may be an indirect indicator of disease state in the ascending aorta because of the effects on downstream flow. More broadly, the current work supports the idea that biofluidic biomarkers can complement and improve upon the information provided by geometrical biomarkers alone.

Despite the fact that previous studies have shown that aortic tortuosity index (ATI) [12] (or related metrics of axial stretch [15],) and maximum diameter (D_max_) [17], [61] are important geometrical biomarkers for ATAA in humans and mice, our results showed only a moderate correlation between ATI (r^2^ =0.21) and Dmax (r^2^ =0.37) with mouse lifespan. Additionally, other mouse models with genetic defects in the elastic fibers show aortic tortuosity without evidence of ATAA [62], suggesting that tortuosity is related to elastic fiber integrity and overall mechanical behavior, but is not a sufficient independent metric for ATAA outcomes. This lack of strong correlation is indicative of a highly complex process that cannot be captured by a single measurement, and it also indicates the importance of individual variations among mice (and, even more so, among humans).

The availability of two- and six-month imaging data allowed consideration of temporal changes in parameters as additional possible biomarkers. We found that change in diameter (ΔD, i.e. growth rate) had a moderate correlation with the lifespan (r^2^ = 0.38, Fig. 8b). This point has received considerable attention as regards human patients (see [63]) with no clear conclusion and challenges because of the slow growth rate of most of ATAAs in humans. It is also notable that although both ΔTAWSS_mean_ and ΔOSI_min_ showed a correlation with lifespan (r^2^=0.75, r^2^ = 0.24, Fig. 7), there was almost no correlation between them (r^2^ = 0.02, Fig. 7), suggesting that the two metrics are complementary. These possibilities require further study in animals and, when possible, in humans. In considering the longitudinal data, we note that the aneurysm had already begun to form in most mice at the first scans that we took at two months. It would be informative to have earlier timepoint data to observe the initial formation of the aneurysm, rather than the growth of the existing aneurysm, although there will be technical challenges and limited imaging resolution in juvenile mice. The data would be particularly valuable in testing and parameterizing *in vivo* models of aneurysm growth [64], [65].

In the current study, some limitations and areas of potential improvement need to be highlighted. The lack of available data for mouse-specific inlet blood flow and outlet boundary conditions (i.e. RCR parameters) presents a limitation on this study. Previous investigations in mice [66], [67] and humans [68], [69] showed that idealized (i.e. parabolic and Wormersley) velocity profile had substantial implications on the WSS distribution in the aortic region close to the inlet. Similarly, it has been shown that hemodynamic quantities are also affected by the choice of the outlet boundary conditions [66], [69]. Additionally, absent detailed regional wall properties, wall thickness and material properties were considered to be constant along the aortic length, ignoring heterogeneities of the arterial tissue that have been shown previously [70], which can potentially lead to skewed stress distribution along the wall. Limitations with respect to the MRI resolution and the small size of the mouse aorta, as well as positioning of the mouse that may affect aortic length measurements, add uncertainties to the calculated biomarker values [71]. We note that, given the relatively small test population and the large number of potential biomarkers, this study should be viewed as demonstrative of technique and as identifying hypothetical biomarkers for future study but not as a definitive determination of the validity of biomarkers. Finally, we used mouse lifespan as a surrogate measure for ATAA outcomes. We euthanized four of the ten mice at 25 months of age and did not do detailed necropsies on the mice with shorter lifespans, so it is possible that these mice died for other reasons than ATAA complications. Despite these shortcomings, the model outputs showed good correlation with mouse lifespan, suggesting that human patients - for whom flow profiles and regional wall properties may not be available - may still benefit from analysis of this type to predict disease outcomes, especially if the large pool of potential biomarkers can be reduced in size by a statistical technique such as principal component analysis. Although this paper is not intended to compare mouse to human studies, we provided a brief discussion of the differences in terms of the current study and its results. Perhaps, most significantly, we used post-mortem, isolated mouse aorta mechanical test data as inputs to the model, whereas if one intended to use a patient-specific model for clinical application, *in vivo* estimates of the properties would be needed. Recent advances in this area [14], [72] offer hope for such data to be readily available in the future. Also, the variability from mouse to mouse, both in terms of aneurysm shape (Fig. 4) and in terms of longevity (Table 1) was dramatic, and the wide range of genetic, environmental, and lifestyle factors in humans implies even more variation. We conclude with the observation that there are countless parameters and combinations of parameters that could be considered as potential geometrical and biofluidic biomarkers. Pulse wave velocity [73], for example, is easy to measure and non-invasive, and it could be estimated from FSI calculations, like the ones performed in this study, or could be used to tune the model’s estimate of the wall properties. Diameter and tortuosity are simple measures, but many other measures (e.g., vessel curvature in the aneurysm, aneurysm volume, or some measure of aneurysm length) could be created and might be meaningful. Machine learning tools to describe the geometry and mechanics of the aorta [74] may provide a platform for more efficiently identifying potential novel biomarkers. Finally, the study was performed using fully-coupled FSI simulations with a nonlinear constitutive equation for the vessel wall, which can be computationally expensive. The Small on Large approach [75], which is significantly cheaper computationally while still capturing much of the vessel behavior, could be considered if the full simulations prove intractable.

## Supplementary Material

1776342_Sup_Info

## Figures and Tables

**Table 1:** Mouse-specific parameters. M = male and F = female in mouse ID. Elastic modulus and unloaded wall thickness are for the ascending aorta.

Mouse ID	Elastic modulus [MPa]	Wall thickness [mm]	Lifespan [months]	ATI
15469_3 F	1.14**	0.142**	12.4	35.4
15398_3 M	0.47	0.142	13.3	52.0
15392_1 M	2.23	0.129	17.8	45.3
15392_2 M	1.16	0.122	18.2	59.5
15466_1 F	0.88	0.125	21.9	53.6
15398_1 M	1.22	0.140	22.4	37.9
15466_3 F	1.59	0.122	25*	39.9
15474_2 F	0.93	0.180	25*	38.8
15474_3 F	0.64	0.162	25*	22.8
15389_1 M	1.14**	0.142**	25*	28.7

* Euthanized after reaching approximately 25 months of age

** Average from the other 8 mice data since mechanical and geometrical data were not available

ATI – Aortic Thoracic Index

**Table 2:** Outflow model parameter values (see Equation (5); based on [24]).

	R_p_ [Pa.s/mm^3^]	C [Pa/mm^3^]	R_d_ [Pa.s/mm^3^]
1. Left subclavian artery	19.58	5.5 × 10^−4^	286.2
2. Left common carotid	44.70	3.23 × 10^−4^	488.0
3. Brachiocephalic trunk	21.55	3.54 × 10^−4^	443.2
4. Outlet	10.30	5.41 × 10^−4^	443.2

**Table 3:** List of geometrical and biofluidic markers

Abbreviation	Description	Range
P_max_	Max Pressure	110-172 mmHg
vMs_max_	Max von Mises Stress	320 – 770 kPa
Re_max_	Max Reynolds Number	143 - 227
OSI_min_	Minimum Oscillatory Shear Index	3.3 × 10^−8^ – 1.2 × 10^−5^
OSI_mean_	Mean Oscillatory Shear Index	0.17 – 0.38
TAWSS_mean_	Mean Time Average Wall Shear Stress	0.9 - 3.42 Pa
TAWSS_max_	Max Time Average Wall Shear Stress	6.1 – 11 Pa
TAWSS_min_	Min Time Average Wall Shear Stress	0.005 – 0.33 Pa
Wo	Womersley Number	1.1 – 2.3
ATI	Aortic Tortuosity Index	22.8 – 59.5
D_max_	Max Aortic Diameter	1.20 – 3.30 mm
ΔP_max_	Change in Max Pressure	0 – 67.5 mmHg
ΔvMs_max_	Change in Max von Mises Stress	10 – 210 kPa
ΔRe_max_	Change in Max Reynolds Number	18 - 75
ΔOSI_min_	Change in Minimum Oscillatory Shear Index	1 × 10^−9^ – 2 × 10^−6^
ΔOSI_mean_	Change in Mean Oscillatory Shear Index	0.012 – 0.063
ΔTAWSS_mean_	Change in Max Time Average Wall Shear Stress	0.09 – 4.3 Pa
ΔTAWSS_max_	Change in Max Time Average Wall Shear Stress	0.064 – 2.3 Pa
ΔWo	Change in Womersley Number	0.07 – 1.2
ΔD_max_	Change in Max Aortic Diameter	0.01 – 0.73 mm
LS	Lifespan	12.37 – 25* months

* Mice that did not die naturally earlier were euthanized after reaching approximately 25 months of age

**Table 4:** HGO model fitting results for *Fbln4^SMKO^* mouse ascending aorta (n = 8)

	Mean	Minimum	Maximum	Standard deviation
C [MPa]	1.77	0.23	4.91	1.53
k1[MPa]	0.43	0.30	10.01	3.37
k2[−]	2.01	2.7e-7	4.30	1.38
*α*[°]	44.95	9.5e-5	89.9	43.0
*κ*[−]	0.17	7.64e-8	0.41	0.16
