# Supplementary material for: Experimental and mouse-specific computational models of the Fbln4SMKO mouse to identify potential biomarkers for ascending thoracic aortic aneurysm
Source: Cardiovasc Eng Technol. Author manuscript; Available in PMC 2023 Aug 1. (PMC9304450; doi:10.1007/s13239-021-00600-4)
Supplement: 1776342_Sup_Info [file NIHMS1776342-supplement-1776342_Sup_Info.pdf]

Supplemental information

S2. Methods

S2.1 Magnetic resonance imaging (MRI)

Heavily T1-weighted axial images were acquired at 187.5 x 187.5 x 500 μm^3 resolution using prospective cardiac and respiratory gating. The images below show an example of the diastolic angiographic images from one mouse. At this resolution, the aorta is typically over five voxels across, making it relatively easy to identify and accurately manually segment.

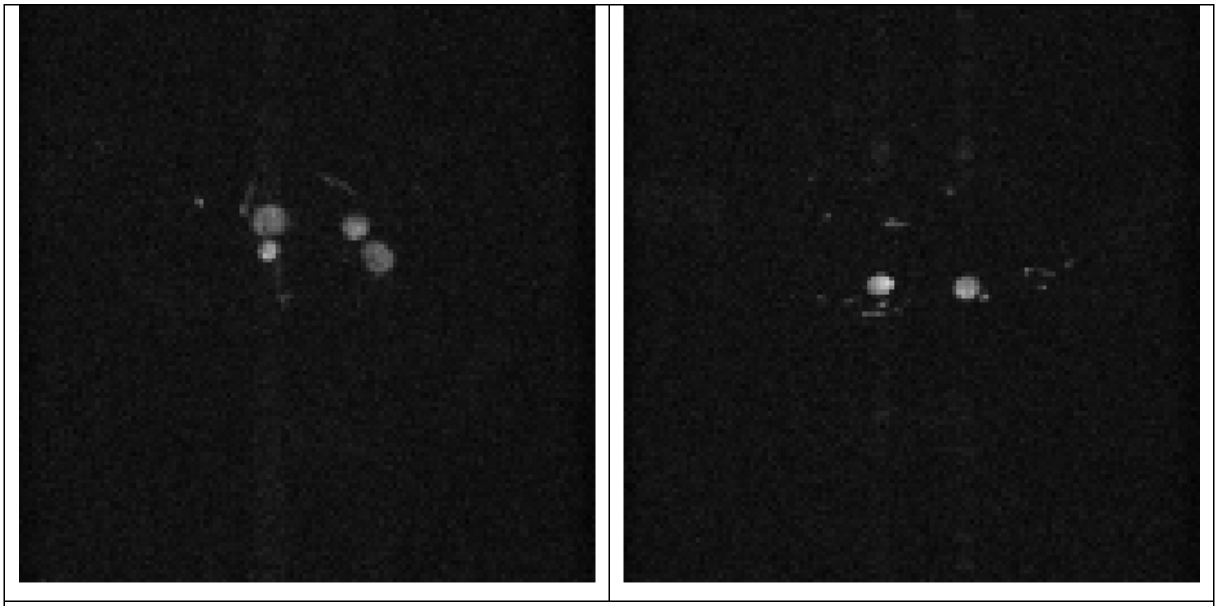

Figure S1: Axial MRI images at 187.5 x 187.5 x 500 μm^3 resolution from above (left) and below (right) the heart. Images are shown after averaging across all diastolic images but without interpolation or smoothing.

For analysis, the images were interpolated to 187.5 x 187.5 x 250 μm^3 resolution to improve the ability to detect changes in vessel orientation. The Maximum Intensity Projection (MIP) image below illustrates the conspicuity of vessels much smaller than the aorta across the mouse thorax and abdomen.

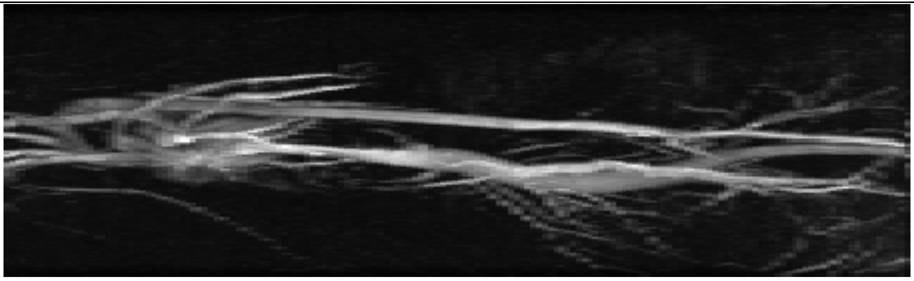

Figure S2: Coronal MIP (Maximum Intensity Projection) diastolic image (cranial to left) after interpolation across slices but without any further processing.

S2.2 Blood rheology

Blood rheology was modeled using the Carreau-Yasuda model. The model parameters were based on previous measurements obtained from human healthy blood. Table S1 shows the comparison between measured viscosity for mice and humans [31] and the Carreau-Yasuda model prediction [30]. We can observe that the Carreau-Yasuda model predicts well the measured viscosity of mouse blood. For the moderate to high shear rate range, which is the range of interest for this study, all three measurements are similar within the confidence interval.

Table S1: Experimentally measured viscosity values for mouse and human blood and the Carreau-Yasuda model predictions for four levels of shear rate.

	WBV (0.7 s^{-1}) [cP]	WBV (2.4 s^{-1}) [cP]	WBV (94s^{-1}) [cP]	PV [cP]
Mice experimental values	13.367 (10.691/16.568)*	10.563 (8.929/12.289)*	4.879 (4.506/5.345)*	1.31 (1.29/1.34)*
Human experimental values	33.487 (26.264/39.584)*	18.529 (15.729/21.493)*	5.996 (5.140/6.938)*	1.24 (1.2/1.31)*
Carreau-Yasuda model	15.12 (10.93/19.18)*	12.79 (7.33/16.48)*	6.54 (5.08/7.36)*	1.2** (1.1/1.31)*

WBV = whole blood viscosity

PV = plasma viscosity

* = confidence intervals for experimental measurements and fitting

** = plasma viscosity is represented by η_∞ in Eq. 6

S3. Results:

S3.1 Mouse-specific FSI simulations

Figure S3 shows the Time Average Wall Shear Stress (TAWSS) distribution along the wall for all ten six-month-old *Fbln4*^{SMKO} mice at the 10th simulated cardiac cycle. A wide range of TAWSS can be observed, but for almost all geometries, low values were observed in the ascending aorta, especially for large aneurysms (Fig S3a and Fig S3d). Interestingly, the opposite behavior was observed for OSI, especially for large aneurysms where large values of OSI were observed along the ascending aorta.

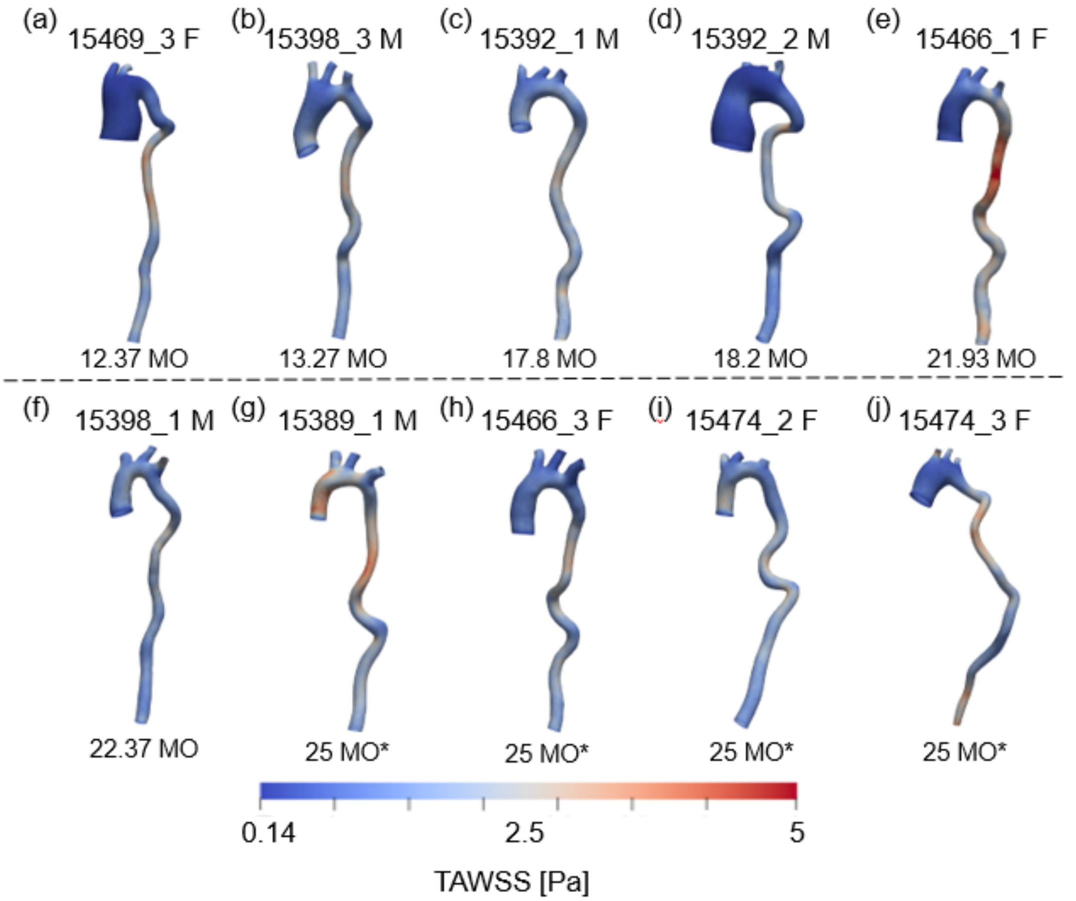

Figure S3*:* TAWSS distribution along the wall for all ten *Fbln4*^{SMKO} mice. Models are organized by increasing lifespan. Mouse ID code and sex (M/F) is given at the top of each model, and lifespan is displayed on the bottom.
